# How to handle the risks of oxygen

**DOI:** 10.1007/s00709-020-01487-z

**Published:** 2020-02-13

**Authors:** Peter Nick

**Affiliations:** Botanical Institute, Karlsruher Institut für Technologie, Karlsruhe, Germany

Life on our planet is driven by solar energy. With the exception of some exotic life forms that still dwell in extreme habitats on the chemical energy derived from geochemical processes, all living beings depend, directly or indirectly, on photosynthesis. However, the benefits provided by this process came with a risk: With time, the product of light-driven water-splitting, oxygen, accumulated in the atmosphere. Oxygen is a dangerous molecule, eager to grip free electrons and giving rise to the even more dangerous reactive oxygen species. Although life succeeded in getting control over oxygen and even exploits this process to generate ATP, the slightest perturbations, as they commonly occur in response to adverse environmental conditions, will lead to the accumulation of these only partially reduced oxygen forms that can cause substantial cellular damage. As illustrated by two contributions to the current issue, the impact of reactive oxygen species shapes the life of both animals and plants:

The contribution by Andrabi et al. (2020) casts a cell biological look upon one of the major death causes, especially in industrialised countries: ischaemic stroke. While the damage caused by disrupted blood circulation has acquired considerable attention, because it seems straightforward that one needs to reinstall blood flow, for instance by surgery or by plasminogen activators to clear the clogged vessel, it is often overlooked that considerable damage occurs during the reperfusion phase, which can be linked with excessive oxidative burst. Excessive reactive oxygen species generated by the mitochondria will then result in a loss of membrane tightness and leakage of metabolites that act as signals for apoptotic cell death. This is especially relevant for the neurological damage often accompanying a stroke episode. Restoration of oxidative balance in the mitochondria would, therefore, be a rewarding target for drug development. The authors set off to describe the system, by which mitochondria maintain their redox homeostasis, to develop then the effect of ischaemia reperfusion stress upon this redox balance, and the role of mitochondrial oxidative burst for cellular signalling. Since, so far, no neuroprotective agent has been identified that is effective in stroke patients, the need to define new targets is evident. So far, pharmacological therapy is confined to thrombolytic agents. Mitochondria represent a promising alternative, and a deeper understanding of their oxidative regulation is mandatory to design lead structures and to assess efficacy.

The contribution by Rudenko et al. (2020) deals with the second organelle which has to handle oxygen, the chloroplast. Here, it is the innate antagonism between fixation of carbon in the Calvin-Benson cycle and the release of oxygen in the water-splitting reaction of photosystem II. The enzyme ribulose-bisphosphate carboxylase (RubisCO) responsible for the transfer of carbon dioxide to a C_5_ sugar moiety can, in the presence of high oxygen levels, catalyse a decarboxylase reaction, which will not only break down the sugar rather than extending it but also produce toxic compounds that have then to be detoxified under consumption of further energy. This so-called photorespiration becomes accentuated under conditions, where high light intensities coincide with a limited water supply, such that the stomata are closed, and the oxygen released by water splitting cannot be efficiently removed. The impact of this reaction for agriculture in times of climate change is evident. Plants have evolved a work-around for this conflict: by binding the carbon dioxide as transportable bicarbonate, they can assign the two reactions (water splitting versus carbon fixation) into different cells. Carbonic anhydrases play a central role for this so-called C_4_ photosynthesis. In flowering plants that have returned to the water, these enzymes are also used to concentrate the traces of carbon dioxide that are dissolved in the water. In addition to these classical functions, carbonic anhydrases are also found in the thylakoid, which is at first sight unexpected and has stimulated the authors to find out what these enzymes are doing during the light reaction of photosynthesis. In their previous work (Rudenko et al. 2018), they had investigated a knockout of this enzyme in *Arabidopsis thaliana* and could find evidence for perturbed photosynthetic electron transport. The underlying mechanisms were now dissected in their current paper. By administering high light stress, where electron transport becomes saturated, such that free electrons cannot be efficiently dissipated and, thus, contribute to oxidative stress, they could show that electron transport through photosystem I was essentially unaltered, while electron transport triggered by photosystem II was significantly increased, while D1, the core protein of photosystem II, became persistent to light-induced decrease. At the same time, carotenoids, pigments that scavenge reactive oxygen species, increase, indicative of a higher level of oxidative stress. Thus, these enzymes that bind carbon dioxide participate in the adaptation of the photosynthetic apparatus to high light-induced oxidative stress.

As different as the two phenomena may appear, they illustrate the all-encompassing selective stringency of oxygen in life forms as different as animals and plants. In the one case, oxygen is consumed as receiver for electrons which allows for production of energy; in the other, oxygen is generated as donor of electrons after water splitting. Both processes have to pass through dangerous intermediate states. Although this task is old and general, there are many ways as to how this task was solved, which tells that it depends on the context how to best handle the risks of oxygen.

## References

[CR1] Andrabi SSA, Parvez S, Tabassum H (2020) Ischemic stroke and mitochondria: mechanisms and targets. Protoplasma, current issue 10.1007/s00709-019-01439-2 31612315

[CR2] Rudenko NN, Fedorchuk TP, Vetoshkina DV, Zhurikova EM, Ignatova LK, Ivanov BN (2018). Influence of knockout of At4g20990 gene encoding α-CA4 on photosystem II light-harvesting antenna in plants grown under different light intensities and day lengths. Protoplasma.

[CR3] Rudenko N, Fedorchuk T, Terentyev V, Dymova O, Naydov I, Golovko T, Borisova-Mubarakshina M, Ivanov B (2020) The role of carbonic anhydrase α-CA4 in the adaptive reactions of photosynthetic apparatus. The study with α-CA4 knockout plants. Protoplasma, current issue 10.1007/s00709-019-01456-1 31784823

